# Biomedical Implementation of Liquid Metal Ink as Drawable ECG Electrode and Skin Circuit

**DOI:** 10.1371/journal.pone.0058771

**Published:** 2013-03-05

**Authors:** Yang Yu, Jie Zhang, Jing Liu

**Affiliations:** 1 Department of Biomedical Engineering, School of Medicine, Tsinghua University, Beijing, China; 2 Key Lab of Cryogenics, Technical Institute of Physics and Chemistry, Chinese Academy of Sciences, Beijing, China; CNR, Italy

## Abstract

**Background:**

Conventional ways of making bio-electrodes are generally complicated, expensive and unconformable. Here we describe for the first time the method of applying Ga-based liquid metal ink as drawable electrocardiogram (ECG) electrodes. Such material owns unique merits in both liquid phase conformability and high electrical conductivity, which provides flexible ways for making electrical circuits on skin surface and a prospective substitution of conventional rigid printed circuit boards (PCBs).

**Methods:**

Fundamental measurements of impedance and polarization voltage of the liquid metal ink were carried out to evaluate its basic electrical properties. Conceptual experiments were performed to draw the alloy as bio-electrodes to acquire ECG signals from both rabbit and human via a wireless module developed on the mobile phone. Further, a typical electrical circuit was drawn in the palm with the ink to demonstrate its potential of implementing more sophisticated skin circuits.

**Results:**

With an oxide concentration of 0.34%, the resistivity of the liquid metal ink was measured as 44.1 µΩ·cm with quite low reactance in the form of straight line. Its peak polarization voltage with the physiological saline was detected as −0.73 V. The quality of ECG wave detected from the liquid metal electrodes was found as good as that of conventional electrodes, from both rabbit and human experiments. In addition, the circuit drawn with the liquid metal ink in the palm also runs efficiently. When the loop was switched on, all the light emitting diodes (LEDs) were lit and emitted colorful lights.

**Conclusions:**

The liquid metal ink promises unique printable electrical properties as both bio-electrodes and electrical wires. The implemented ECG measurement on biological surface and the successfully run skin circuit demonstrated the conformability and attachment of the liquid metal. The present method is expected to innovate future physiological measurement and biological circuit manufacturing technique in a large extent.

## Introduction

Electrocardiogram (ECG) reflects the electrical activity of the heart, which is an essential physiological parameter for both clinical diagnostics and health monitoring. To detect the weak signal of ECG non-invasively, the electrode is usually stuck onto the skin of limbs and chest for better measurement.

There are various ECG electrodes with different materials and shapes, such as flat metal electrodes, suction electrodes and disposable button electrodes etc. However, these conventional electrodes may encounter troubles such as coupling performance, contact comfortableness, manufacturing complexity and cost. The flat metal electrodes are rigid plates that clamp the limbs and the suction electrodes are small evacuated cups that suck tightly to the skin. Both kinds of electrodes are commonly used on the ECG instruments in clinics and hospitals. They usually have poor coupling performance with the skin and high signal noise that has to be overcome with electrolytic gel. And such electrodes may cause discomfort and even hurt to human body due to their mechanically rigid fixing. The disposable button electrodes are mainly used in portable ECG monitor devices with electrolytic gel for conduction enhancement and Ag/AgCl as electrode material to pursue better performance [1]. However, the structure of the button electrodes is quite complicated, including the electrolytic gel, Ag/AgCl disk, steel button cap, adhesive plaster, sealing plastic and paper covers, thus making it more expensive, especially when considering that silver is a noble metal. And the sticking plaster sometimes can take off the hair, causing pain and even inflammation in the hair sac. In order to find out alternative electrodes or ways with better features, various strategies have been investigated. For example, Cauwenberghs' group at UCSD have managed to implement the non-contact electrode to improve comfort degree, which explores a new approach of monitoring ECG and EEG signals wirelessly [2]–[4]. Another attractive idea is the so-called epidermal electronics, which is developed by Rogers' group at UIUC [5]. It is a conformal device attached to the skin like a tattoo with stretchable electrical sensors, which could measure physiological variables such as ECG, EEG and EMG.

A latest discovery in Ga-based liquid metal alloy inspires the exploration [6], [7], which can possibly change the form of the electrodes, even the bio-electrical detection modality. Here, we would propose to use the Ga-based liquid metal ink as bio-electrodes and skin electrical circuit material. Such alloy owns many unique merits such as low melting point, direct printability, good electrical conductivity and mechanical flexibility, biocompatibility, low cost and so on, which overcomes the shortages of the conventional materials in a large extent.

In the present work, we demonstrated the feasibility and advantages of using liquid metal alloy as bio-electrical sensors, and accomplished a series of conceptual experiments to evaluate its performance as ECG electrodes. The alloy contacts the skin well and has relatively low polarization voltage, which ensures the good signal quality. The results indicated that the Ga-based liquid metal could serve as a favorable electrode material for detecting ECG. Though still in its primary stage, the liquid metal ink electrode opens a wide area of new applications on human healthcare. Meanwhile, we also managed to draw a basic printable circuit on the skin using the liquid metal ink, which illustrated the innovative idea of conformable biological circuits. Together with the ECG testing with liquid metal electrode, the experiment indicated that it is promising to print or “draw” a circuit on the skin to achieve the goal of low-cost and point-of-care physiological detection.

The adopted liquid metal ink is safe, inexpensive and easy to use. Hence the alloy, as a new form of bio-electrical sensor and printable skin circuit material, is fully compliant with pervasive healthcare, which generally requires low cost and convenient practice. It can be printed and erased easily, making it much easier to realize the self-service of bio-electrical detections and mapping. With good wetting ability, the present metal ink enables to make the biomedical detection system on the skin, which may significantly shape the printable or “drawable” bio-electronic technology in the near future.

## Materials and Methods

The currently used liquid metal ink is composed of gallium or gallium-indium alloy with oxide. The melting point of gallium is around 30°C with the nature of supercooling, which keeps it in liquid state at room temperature. By vigorously stirring the liquid metal under strictly controlled conditions, the quantity of oxygen in the material will increase with the stirring time, together with increment of the viscosity and the electrical resistivity of the alloy. Compared with the conventional gallium simple substance, the new material reaches a good balance, which has both good electrical properties and attaching ability. The Ga-based liquid metal in our experiments possessed an oxygen concentration of 0.34%.

**Figure 1:**
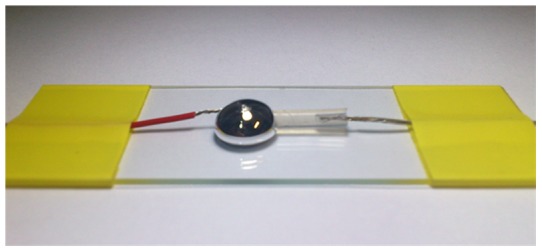
The physiological saline is sealed in a plastic tube. The anode (Pt probe) sticks into the tube and the cathode (Pt probe) into the liquid metal droplet. When the tube and the droplet contact, the polarization voltage emerges and is recorded through the probes.

Through directly printing the liquid metal ink on the skin of either animal or human body with a paintbrush, the alloy layer becomes the simplest electrode and interface for recording electrical signal in human body. Thus we measured two basic properties of the liquid metal electrode at room temperature: the impedance and polarization voltage. When the electrode and the electrolyte solution contact, an electrical couple layer is formed at the boundary, causing about the contact voltage and the polarization phenomenon [8]. In Figure 1, we took the physiological saline as a simulated tissue fluid and measured the voltage with the Ga-based liquid metal at room temperature. Agilent 34970A data acquisition/switch unit was used to record the voltage and the probe material is platinum. For impedance measurement, the liquid metal ink was filled in a 40 cm long plastic tube, which has an inner diameter of 1 mm. And we adopted the SRS Model SR780 2-Channel Network Signal Analyzer for impedance measurement.

**Figure 2:**
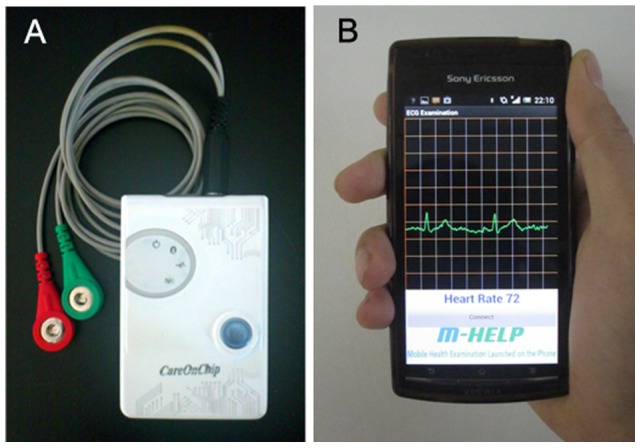
A portable system for ECG monitoring on the mobile phone. (A) The miniaturized wireless ECG module; (B) The ECG monitoring displayed on the mobile phone.

In order to provide practical verification, we designed the following conceptual experiments: the liquid metal ink electrode was used for testing the real electrocardiogram of rabbit and human, respectively. The experiments were operated with a miniaturized module (Figure 2A) developed on the mobile phone (Figure 2B) to monitor the ECG wave, which is compatible with both conventional Ag/AgCl button electrode and the liquid metal ink. The acquired data can be transmitted to a smart phone (Sony Ericsson lt15i, Android 4.0 OS) via Bluetooth and displayed on the mobile phone screen (Figure 2). The ECG module has two wires for conducting the differential biopotential into the filter circuit. There is a metallic button connector at the end of either wire, which is used for the button electrode to plug in and it can be seen in Figure 2A. The liquid metal ink was printed on the skin surface with a paintbrush, and the button connectors were directly attached to the alloy when using it for acquiring ECG signals. Though it is not a rigid connection, the conformability and wettability of the liquid metal ensures its good contact with both the skin and the metallic button connector, i.e. the module.

In addition, the good wettability and electrical conductivity indicates that the liquid metal ink is not only a favorable bio-electrode, but also an ideal alloy for making conformable circuits on the skin or any other surfaces. Hence we managed to “draw” a basic electrical circuit in the palm using the ink in later section. With batteries hold in hand, the circuit's work status is displayed with four surface mounting LEDs attached in. This trial can be seen as a symbol of more sophisticated biomedical applications with the material in the near future.

In the present experiments, rabbit was chosen as the test animal. The first author of this article volunteered as participant in the skin ECG test with a written consent kept in the lab. The Ethics Committee of Tsinghua University, Beijing, China has specifically approved the present study under contract [SYXK (Jing) 2009–0022].

## Experimental Results

### Impedance and Polarization Voltage

**Figure 3:**
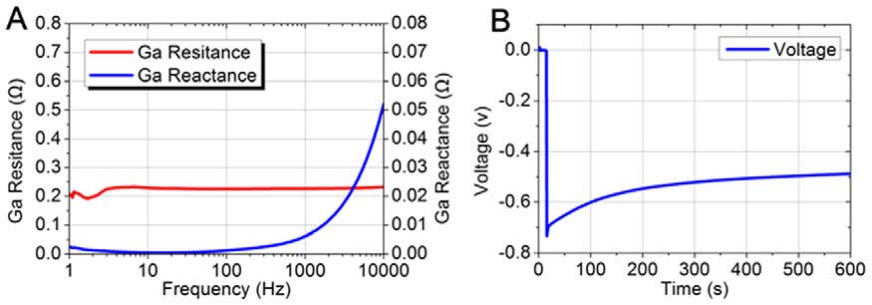
The impedance and polarization voltage of the Ga-based liquid metal ink. (A) The resistance and reactance curve from 1 Hz to 10 kHz for the liquid metal in a 40 cm long tube with a diameter of 1 mm; (B) The polarization voltage curve when the liquid metal contacts with physiological saline.

Figure 3A shows the resistance and the reactance as a function of frequency from 1 Hz to 10 kHz. The calculation result of the average resistance is 0.225 Ω, representing an electrical resistivity of 44.1 µΩ·cm at room temperature. The resistivity of the alloy is higher than common conductive metals such as gold (2.2 µΩ·cm), silver (1.6 µΩ·cm), copper (1.7 µΩ·cm) or pure gallium (25.8 µΩ·cm), yet much lower than a more famous liquid metal – mercury (95.8 µΩ·cm) [9], [10]. It means while gaining a better wettability, the ink keeps at relatively low resistivity as a liquid conductor. On the other hand, the reactance which is correlated with the shape shows a character of low inductance of about 50 µH in the form of a straight line. Since the frequencies of most biomedical signals are below 10 kHz, the influence of reactance can be negligible and hence the resistance remains the major determinant of the impedance. Considering the liquid state of the metal, the measurement indicates that such ink is a promising choice for constructing conformable biomedical electronics.

The polarization voltage is depicted in Figure 3B. When the metal and the saline contact with each other, the voltage “dives” to −0.73 V. After that, the value goes up slowly according to the time-voltage curve. Yet the magnitude stays higher than 500 mV in a few minutes.

The voltage between different phases is determined by multiple factors, including the metal's inner potential, categories and concentrations of the ions in the electrolyte, the temperature and the electrostatic charge adsorption, respectively. Though some of these factors drift rapidly in the physiological environments and can hardly be accurately measured, the simulation experiments still reveal the trend and scale of its polarization.

### ECG Tests on Rabbit and Human

**Figure 4:**
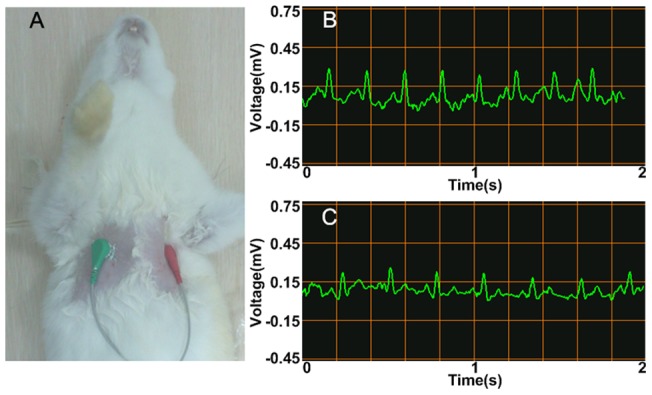
ECG test and results on a rabbit with normal and liquid metal electrode. (A) ECG test on a rabbit; (B) Results using button electrode; (C) Results using liquid metal electrode.

**Figure 5:**
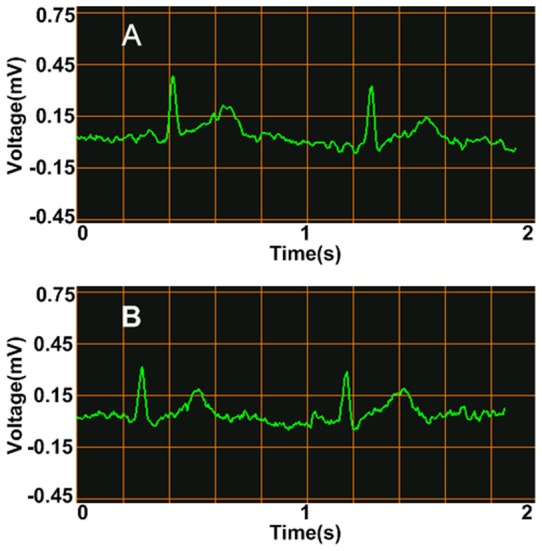
ECG test results on human. (A) Results using button electrode; (B) Results using liquid metal electrode.

Since the liquid metal ink possesses good conductivity and viscosity, we were able to use it as ECG electrode. The conceptual experiments of measuring ECG with liquid metal ink electrodes were carried out on both rabbit and human respectively, and conventional button electrodes were also adopted for a comparison. All the ECG data were acquired by a miniaturized ECG module, which was wirelessly connected to a mobile phone. The ECG waveform can be recorded and displayed in the phone screen in real time. Since the ECG wave amplitude of a rabbit is relatively small, its ECG recording was operated with the lead in the chest, which is displayed in Figure 4A, while the tests on human measured voltage between left hand and right hand (Lead I). Figure 4B and 4C show the consistency of the ECG waveforms that are separately recorded using the button electrodes and the liquid metal ink on the identical rabbit, and Figure 5 displays the similar results with the records of human ECG. Both results have shown that the qualities of the recorded ECG signals were almost the same, which indicates that the liquid metal is competent as a new kind of bio-electrode.

As is well known, it is quite hard to apply ECG test on small animals because of their sizes. The conventional electrodes have to include alligator clips and probes sticking through the skin. This may cause lesions to the animals, while the liquid metal ink overcomes these disadvantages. Thus besides its convenience, the liquid metal ink electrode also brings better humanity, to which the rabbit experiment is a good proof.

### Drawable Skin Circuit

**Figure 6:**
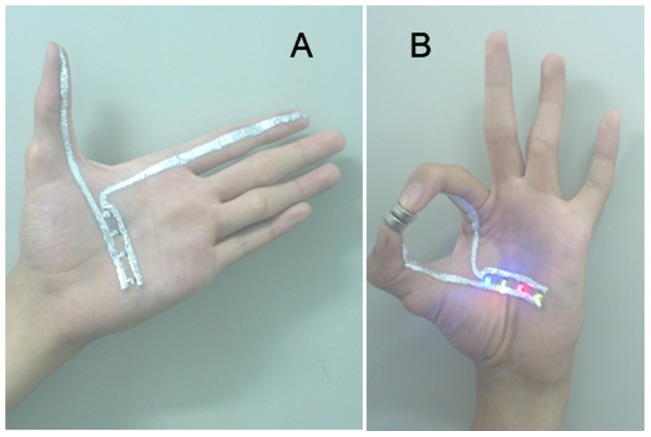
The skin circuit drawn in the palm. (A) The basic circuit drawn in the palm; (B) The circuit works when the batteries are gripped and the hand forms a gesture of “OK”.

It is a basic circuit with battery, resistors and LEDs, but the wires are made of liquid metal inks, which are simply drawn on the skin with paintbrushes. Either to draw or to modify the circuit is an easy job because it is just like painting and erasing with normal ink, where the place of canvas has been taken by the palm. Regardless of the veins or the movements, when the batteries are gripped between the thumb and the index finger, the loop closes and everything goes “OK” with the colorful lights, which is presented in Figure 6. While the circuit is working, neither thermal nor electrical stimulation can be sensed from the ink. And though the trace becomes drier and caused a little feeling of discomfort to the skin, yet it can be easily washed off with water and soap, leaving no other perceivable irritation.

In a broader view, the experimental results of liquid metal ink as both bio-electrode and skin circuit have strongly illustrated the feasibility of combining liquid metal ink with mobile health systems. On the purpose of providing a point-of-care access to healthcare, the ink is easygoing to be drawn or printed as electrodes or functional patterns.

## Discussion

The liquid metal ink shows great virtues that no other conventional material possesses, and the conceptual experiments demonstrate its practical value in biomedical measurement. But before the actual use, there are still many issues need to be clarified.

### Safety Issue

The characteristic of the liquid metal ink that people may pay special attention to is about its safety. According to a systematic review on gallium and galinstan hazards, the metal and its alloy have very low vapor pressures, meaning that a small amount of the material would not evolve any metal vapor. And the gallium and galinstan do not react with normal electrical insulation, nor do they corrode copper or stainless steel at room temperature. Gallium is insoluble in water and thus can hardly be absorbed through the skin. Long period inhaling of gallium oxide in large quantity may cause lung damage. However, naturally the gallium oxide produced forms a layer instead of becoming dust in the air. There is also report of gallium chloride-containing complexes poisoning, yet case like this is rare even in the laboratory [11]. Only one thing that needs caution is that eye contact or injection through the skin (including contact through wounds) should be avoided. Thus unless contacting the material in long terms or large doses, it is safe for normal use with small amount [12]. There are also other supports on gallium safety: gallium alloy has been used as restorative material in dentistry [13] and Ga-67 scintigraphy is an important body inside medical imaging method [14], [15]. And it is easy to remove the liquid metal by spraying medical alcohol solution, whose evaporation cools the material to solid form and much easier for separation with the substrates.

### Electrical Properties of the Ink

The impedance and polarization voltage are among the major concerns when evaluating a material's performance as electrode. The measurements of both parameters illustrate that the liquid metal ink is capable of serving as electrode and circuit material.

Though its resistivity is higher than the normally used metals such as copper or silver, yet it is still acceptable because in most cases this scale of resistance is negligible. But it is obviously not suitable to be an electrical heater unless there are some other energy sources, such as microwave, laser beam and even a contact heater. The reactance varies in different situations such as the change of shape, length and thickness. Thus it is possible to make this use into a sensor of motions, especially in the area of joints and muscles. And the reactance should also be thought about carefully when designing antennas or circuits that involved in high frequency signals.

Though we do not investigate the relative electric potential of the material, the experiment as have been done already reflected the polarization. The magnitude of the contact voltage stays at a high level and it is an unstable but always existing source of noise when recording biopotentials. As a contrast, the polarization magnitude of a button electrode with electrolytic gel is usually smaller than 0.1 V, which means that the ink may not perform as good as an ordinary medical electrode. However, the waveform in both ECG experiments illustrates that the liquid metal is still an acceptable bio-electrode. Thus a device with well designed signal filter and reasonable common mode rejection ration (60dB∼80dB) is capable to fulfill the request.

### Conformable Circuits Technique

The good wettability is another important character the liquid metal ink owns. The skin circuit experiment has verified that it is possible to make an electrical circuit on the rough surface like the skin, which can be easily drawn or erased. As a comparison, it is far more convenient and efficient than the current printed circuit board (PCB) technique, which is rigid and has to make a new one in case of any changes.

This may lead to the unique technique of conformable circuit, which means not only its product is conformable, but also the manufacture of the circuits is adapted to any surface. And the technique can still be divided into two parts: one is electrical circuit drawn by hand or with simple tools, which adequately supports free creation; another is liquid metal printer, which is suitable for precise circuit and industrial production. Both parts can provide supplementary to each other, too. In order to realize multiple functions, it will be valuable to do research in making liquid metal components such as antenna and IC chips, and studies on the embedded batteries or power supply will be important as well. In either case of liquid metal circuit manufacture, the biomedical applications will be an extremely important part of the technique, due to its safety, convenience, low cost and of course flexibility.

### Biomedical Application with Liquid Metal

**Figure 7:**
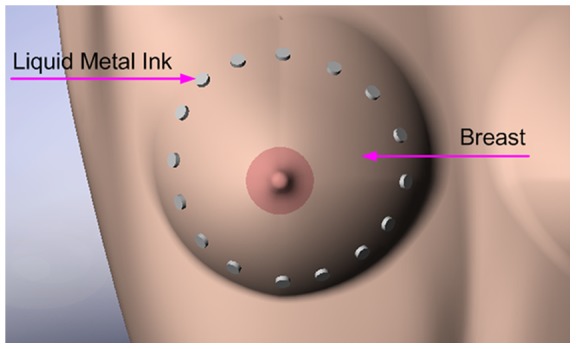
A 3D model of using liquid metal ink as breast EIT electrodes.

This is the first trial of using the liquid metal as a physiological electrode for ECG detection, which is a good example of the material's biomedical roles. It is specifically suitable for electrical impedance tomography (EIT) [16]–[18] due to its electrical property and conformability. Figure 7 presents a sketch of a 16-electrode circle array surrounding the breast for EIT. Compared with conventional approaches, the liquid metal ink electrodes provide better contact and more comfortableness with no need of pressing the breast tightly. And similarly it is a prospective electrode in EIT pulmonary monitoring for the children or the new born. The liquid metal ink electrode is also suitable for various bio-electrical (3D) mapping because the electrodes can be drawn at any area and in any shape, including EIT, ECG and EEG. Clearly, the liquid metal electrode can be extended to defibrillator in emergency first aid, too.

The contact voltage can be applied in other ways as well. For example, it has been published that using the liquid metal and its matching metal can help make thermal couples, which is a prospective application for body temperature mapping in the future [19].

Another attractive approach is the functional circuit made by the liquid metal on body surface. For examples, we can use the material to build coils as a signal transmitter for sending out the physiological data acquired in the human body. And the coil can be an energy receiver charged by microwave to realize localized or even whole body heating as well, which is an important method for non-invasive tumor treatment. What's more, we may associate the gallium electrode or circuit with its role in chemical tumor treatment [20] or antimicrobial [21], thus to find its potential value in disease treatment and reasonable ways of releasing it. All of the ideas represent different branches that provide plenty of contents for research, and the liquid metal will make a large difference in biomedical applications.

## Conclusions

This work performed the first experiments to demonstrate ECG measurement based on liquid metal printed on the body surface. And the skin circuit also successfully verified the new electrode's conformability and good attachment. As a natural conductor, the liquid metal ink owns unique multi-side properties such as electricity, wettability, printability and safety, which guarantees it a perfect material for quite a few emerging biomedical practices. The present method opened possibilities for a wide variety of physiological measurement and health care. It is expected to bring promising impacts in both biomedical engineering and circuit manufacturing technique in the coming time.
